# Osteopontin, asbestos exposure and pleural plaques: a cross-sectional study

**DOI:** 10.1186/1471-2458-11-220

**Published:** 2011-04-08

**Authors:** Giuseppe Mastrangelo, Gianluca Marangi, Maria N Ballarin, Silvia Michilin, Aline SC Fabricio, Flavio Valentini, John H Lange, Ugo Fedeli, Luca Cegolon, Massimo Gion

**Affiliations:** 1 Department of Environmental Medicine and Public Health, Padua University, Padua, Italy; 2 Occupational Health Service, Primary Care Trust 12, Venice, Italy; 3 ABO Association, Regional Centre for the Study of Biological Markers of Malignancy, Department of Clinical Pathology, Local Health Authority 12, Venice, Italy; 4 Occupational Health Service, Occupational Health Service, Primary Care Trust 13, Dolo (Venice), Italy; 5 Envirosafe Training and Consultants, P.O. Box 114022, Pittsburgh, PA 15239-0522, USA; 6 Regional Epidemiological Service, Veneto Region, Castelfranco Veneto (TV), Italy; 7 Imperial College London, School of Public Health, St. Mary's Campus, London, UK; 8 Regional Centre for the Study of Biological Markers of Malignancy, Department of Clinical Pathology, Primary Care Trust 12 - Venice, Italy

## Abstract

**Background:**

Osteopontin (OPN) is a plasma protein/cytokine produced in excess in several malignancies. In a recent study OPN was reported as being related to the duration of asbestos exposure and presence of benign asbestos-related diseases; however, it was unclear whether this protein was an indicator of exposure or effect.

**Methods:**

In 193 workers, 50 with pleural plaques (PP), in whom different indicators of past asbestos exposure were estimated, OPN plasma levels were assessed using commercial quantitative sandwich enzyme immunoassays according to the manufacturer's instructions.

**Results:**

Osteopontin increased with increasing age and several aspects of asbestos exposure, without differences related to the presence of pleural plaques. At multivariable regression analysis, the explanatory variables with a significant independent influence on OPN were length of exposure (positive correlation) and time elapsed since last exposure (positive correlation).

**Conclusions:**

Since asbestos in lung tissue tends to wane over time, OPN should decrease (rather than increase) with time since last exposure. Therefore, OPN cannot be a reliable biomarker of exposure nor effect (presence of pleural plaques).

## Background

In the absence of quantitative exposure data, asbestos exposure could be retrospectively evaluated. According to the job-exposure matrix (JEM) approach, the workers are divided into homogeneous groups, based on combinations of plant, work area, job title, historical period; the available industrial hygiene measurement series are then related to the same groups. The main limitations of this approach are that: (i) trends over time are often unknown; and (ii) the inter-individual variations within "homogeneous" groups - i.e.: same job title - can sometimes be more relevant than those between groups [1].

In the job-specific modules (JSM) approach, the most significant factors capable of affecting the exposure intensity are first identified; their relative importance is then assessed, based on the available industrial hygiene historical data, or the engineering evaluation of tasks/operations, or both of them. The main limitations of the JSM approach are: (i) the relative importance of the various determinants may prove difficult to be assessed; (ii) concordance among experts about this item may be poor; (iii) the quality of the available information about the determinant(s) may vary among the study subjects, so that for someone all the details of the tasks involved by their job may be carefully described, while for someone else the job title is barely known [1].

Osteopontin (OPN) is a plasma protein/cytokine that is produced in excess in malignant mesothelioma and cancer of the lung, breast, colorectal, stomach, and ovary [2-4]. Pass [5] reported that OPN was related to the duration of asbestos exposure and presence of benign asbestos-related diseases. However, workers with pleural plaques and asbestosis also had a longer duration of exposure; it was therefore unclear whether OPN was an indicator of exposure or effect. With the aim of testing a new procedure possibly useful in surveillance of past asbestos workers, the present investigation assesses the relationship between plasma OPN levels and asbestos exposure or the presence of pleural plaques (PP), in asbestos workers formerly examined within the framework of a post-occupational medical surveillance program supported by the Veneto Region and the Italian Ministry of Health [6,7].

## Methods

### Study population

904 workers previously exposed to asbestos - already examined at the Occupational Health Service (OHS) of the Primary Care Trust (PCT) 12, Venice, Veneto Region (Italy) - were stratified by cumulative exposure and PP and 30% of them were extracted from each stratum with a random sampling procedure. The final sample included 263 subjects, 54 with and 209 without PP. These subjects received a letter of invitation and respondents were phoned one or two weeks later to fix an appointment for a medical examination. One hundred and ninety two out of 263 workers (73%) agreed to be examined. Since the original number of subjects with PP was only 54, in order to avoid small numbers, 8 additional newly examined workers with PP were included in the study, totalling 200 examined subjects.

After providing written informed consent, subjects were examined by occupational physicians, using the same protocol for collecting clinical and occupational histories and taking blood samples. Incidental findings were discussed with the patients and their primary care physicians and, where appropriate, referred for specialist evaluation. Information on smoking habits was collected and smoking cessation was recommended and facilitated for all patients.

### Ethical Considerations

Approval by the local ethics committee was not required since the medical surveillance of workers formerly exposed to asbestos was a mandatory activity (Veneto Region Decree Law n. 5094, Dec 28^th ^1998; and Veneto Region Directorate Decree No. 48 of 20/12/2006, that included collection of blood samples in the protocol of medical surveillance).

### Assessment of historical asbestos exposure

We used an internationally established questionnaire that allows the estimation of past asbestos exposure using JSM [1]. On the basis of defined scales, examiners scored the determinants of exposure: raw materials used (with fibre content and friability); jobs undertaken (specified in terms of mechanical disturbance applied to materials through the tools used by the worker); and factors modulating exposure (particle emission speed, source surface, presence of localized air exhaust systems, dimension and physical characteristics of the rooms, etc.). Through direct knowledge or literature data describing historical exposure levels in different jobs/tasks, a reference database was separately collected. Using this a priori knowledge, or integrating evaluation from all of the above scores, an exposure intensity was attributed. Lastly, a semi-quantitative estimate of cumulative exposure was made by multiplying intensity (concentration), frequency (percent of the working time spent at a certain exposure level) and length of exposure in years, and by summing up as many products as were necessary to take into account the different jobs undertaken. The interviewers were trained in the use of the questionnaire in order to minimize the information bias.

### Plasma samples and Osteopontin ELISA assay

Blood samples (12 ml) were collected by forearm venipuncture in BD Vacutainer sterile tubes containing K_2_-EDTA and processed within maximum 2 hours. The blood was centrifuged at 1,500 g for 20 minutes at room temperature. Plasma was then collected, aliquoted in sterile Safe Lock tubes, and stored at -25°C until further analysis. Repeated freezing and thawing cycles were avoided.

Plasma OPN was analyzed using an Osteopontin TiterZyme ELISA kit (Assay Designs, Ann Arbor, MI) according to the manufacturer's instructions and results were expressed in ng/ml. All samples were coded for a blinded analysis, and each plasma was determined in duplicate. Quality controls were analyzed in every plate. The analytical validity of the assay was also independently confirmed, and optimal results in terms of sensitivity, precision and recovery were obtained. The intra-assay precision was 6.5% at 24 ng/ml and 3% at 56.7 ng/ml, with analytical sensitivity of 1.78 ng/ml. Moreover, test of linearity (four dilutions from 1:2 to 1:16) and recovery from spiked samples gave acceptable results (mean recovery: 102% and 95.5%, respectively). All assays were carried out in a single session in the same laboratory (Regional Centre for the Study of Biological Markers of Malignancy, PCT 12, Venice, Veneto Region, Italy).

### Statistical analysis

The mean, standard deviation (SD), median, 25% and 75% percentiles, and the results of Shapiro-Wilk test for normality were calculated in two groups of subjects (with or without PP) for OPN, age, time elapsed since first exposure (TSFE), time elapsed since last exposure (TSLE), length of exposure, peak asbestos level (highest asbestos exposure for any job held), and cumulative asbestos exposure. The two groups were then compared by calculating the non-parametric Wilcoxon rank-sum test statistics and the corresponding p-values.

Smoking was coded in three classes: non-smokers, ex-smokers (those who had given up smoking for at least one year), and current-smokers (if he/she regularly smoked at least one cigarette per day or a pipe or one cigar a week for at least a year or for shorter periods that added up to 12 months). The comparison of smoking classes between the two groups (with or without PP) was performed using the chi-square (χ^2^) test.

Several models of linear regression (and several bivariate scatter diagrams) were built, separately in workers with and without PP, where the dependent variable was always OPN and the independent variable was age or TSFE, TSLE, duration of asbestos exposure, peak exposure, cumulative exposure, smoking classes. To test whether the coefficient estimated over the group without PP was equal to the coefficient estimated over the group with PP, the Chow test statistics and the corresponding p-values were calculated.

Data of both groups were pooled together and OPN was regressed against age and asbestos exposure indices at univariable and multivariable linear regression analysis.

To convert OPN into a normally distributed variable, the inverse transformation (1/x) was chosen because it minimized the χ^2 ^test for departure from normal distribution. The normal distribution of the transformed variable was then checked using a Q-Q plot (quantiles of 1/OPN against the quantiles of the normal distribution), a graph of kernel density estimates (Epanechnikov kernel function) against normal density, and the Shapiro-Wilk normality test.

At multivariable analysis, two models of linear regression were fitted where 1/OPN was the dependent variable and the above indicators acted as predictors. In Model 1, the associated risk factors were identified with the backward stepwise selection of predictors using 0.05 as criterion. In Model 2, age was introduced into the former model. In order to obtain more interpretable results, other models of multivariable linear regression were fitted where the dependent variable was OPN and the predictors were the same as the above. Normality of the residuals was checked using a Q-Q plot, a graph of kernel density estimates, and the Shapiro-Wilk normality test.

According to Checkoway [8], the matrix of correlation coefficients "r" (and the scatter plot matrix) between all possible pairs of the independent variables was used to estimate collinearity (linear relationship between two predictors). Furthermore, when comparing a crude (univariable regression) with an adjusted (multivariable regression) estimate, the inflation of the standard error of a regression coefficient was used to check on the degree of multicollinearity (when more than two variables are involved), while the change in the regression coefficient was used to evidence a confounding effect.

All statistical analyses were carried out with STATA 10 (Stata Corporation, College Station, Texas, USA).

## Results

Seven subjects with missing values were discarded; the analysis refers to 193 subjects, 50 with and 143 without PP.

The key characteristics of subjects, categorized by PP, are displayed in table 1. The distribution was not normal for all variables except TSFE in workers without PP, while it was normal for all variables except cumulative exposure in workers with PP (Shapiro-Wilks test, data not shown). The non parametric Wilcoxon rank-sum test provided statistically significant differences between groups for age, TSFE, cumulative and peak exposure, but not for OPN, TSLE, and length of exposure. The median of TSLE (14 in the first and 15.5 years in the second group) was close to the time elapsed - 16 years - between 2008 (year of observation) and 1992 (ban of asbestos in Italy). The workers in the first quartile of TSLE were probably those employed in asbestos decontamination in industry and housing after 1992. The top quartile of TSLE included workers no longer exposed to asbestos from at least 18 years. Although length of exposure was quite long, most subjects were not in an advanced age. The ban of asbestos in 1992 combined with a high length of exposure resulted in high values of TSFE. It can be seen that cumulative exposure in workers with PP largely exceeded that of workers without PP. This difference could be explained by a higher peak exposure, given that length of exposure was fairly similar in both groups.

**Table 1:** Mean and standard deviation (SD), median and quartiles (25-75 centiles) of the key characteristics and p-value of the Wilcoxon rank-sum test comparing two groups, with or without pleural plaques (PP)

Key characteristics	Without PP (N = 143)		With PP (N = 50)		p-value
		
	Mean (SD)	Median (25-75 centiles)	Mean (SD)	Median (25-75 centiles)	
					
Osteopontin (ng/ml)	53.89 (19.57)	49.53 (42.50-57.64)	55.25 (20.86)	50.87 (40.78-66.22)	0.872
					
Age (years)	62.90 (6.40)	61.94 (58.12-67.37)	65.78 (6.05)	64.71 (61.86 -68.33)	0.005
					
Time since last exposure (years)	13.71 (6.69)	14 (8 -18)	15.36 (5.50)	15.5 (12-20)	0.067
					
Time since first exposure (years)	42.20 (6.92)	42 (37-47)	45.12 (6.38)	45 (40-50)	0.010
					
Length of exposure (years)	26.24 (6.97)	27 (22-30)	27.70 (5.67)	28 (25 - 29)	0.170
					
Cumulative exposure (ff/ml) × years	166.13 (120.2)	155.53 (44.79-299.2)	237.52 (102.3)	298.08 (155.5 -301.1)	0.001
					
Peak exposure (ff/ml)	56.49 (59.7)	13.5 (13.5-135)	88.32 (60.3)	135 (13.5 -135)	0.002

Non smokers, former smokers and current smokers were, respectively, 29%, 60% and 10% in the group without PP and 14%, 20% and 66% in workers with PP; there was a borderline level of significance (p-value = 0.044) at the χ^2 ^test.

In former and current smokers, respectively, starting age of smoking was 16.9 (SD = 3.7) and 18.6 (SD = 5.1) years, age at smoking cessation was 40.0 (SD = 10.9) and 60.3 (SD = 6.1), number of cigarettes/day was 20.3 (SD = 13.5) and 14.1 (SD = 6.8), and the number of pack-years was 23.1 [= (40.0-16.9) × (20/20)] and 29.2 [= (60.3-18.6) × (14/20)].

**Figure 1:**
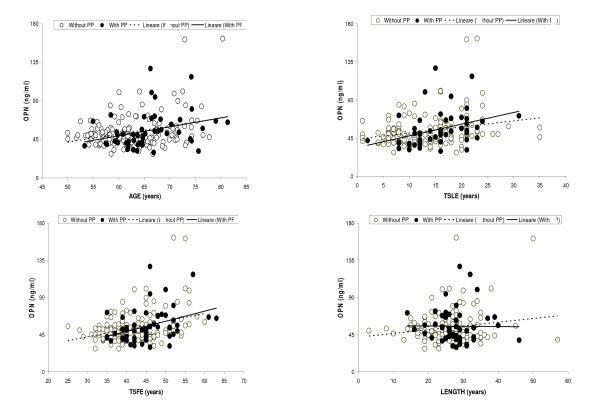
**Scatter plot of Osteopontin (OPN) versus age, time elapsed since first exposure (TSFE), time elapsed since last exposure (TSLE) and length of exposure, in workers with and without Pleural Plaques (PP), separately**.

**Figure 2:**
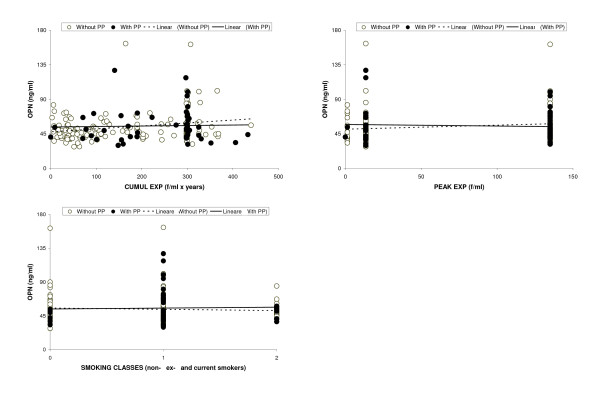
**Scatter plot of Osteopontin (OPN) versus cumulative exposure, peak exposure and classes of smoking, in workers with and without Pleural Plaques (PP), separately**.

Table 2 and figure 1 and 2 show that the trend of OPN over age or each aspect of exposure to asbestos fibres (TSLE, TSFE, length, cumulative and peak exposure) was not significantly different in relation to the presence of PP. Since smoking and peak exposure are not continuous variables, the regression coefficients could be interpreted as a linear trend across ordered categories.

**Table 2:** Linear regression coefficients (b), standard error of the coefficient (SE(b)) and p-value of the Chow test statistics, comparing two coefficients estimated over two groups, with or without pleural plaques (PP)

Predictor variables ^§^	Without PP (N = 143)		With PP (N = 50)		p-value
		
	b	SE(b)	b	SE(b)	
Age (years)	0.93	0.25	1.05	0.47	0.88
Time since last exposure (years)	0.71	0.24	1.35	0.51	0.52
Time since first exposure (years)	0.94	0.22	1.23	0.44	0.74
Length of exposure (years)	0.45	0.23	- 0.03	0.53	0.95
Cumulative exposure (ff/ml) × years	0.04	0.01	0.01	0.03	0.48
Peak exposure (ff/ml)	0.05	0.03	-0.02	0.05	0.46
Smoking classes	-1.67	2.72	1.16	5.14	0.50

^§ ^The dependent variable is Osteopontin in all models of linear regression analysis.

**Figure 3:**
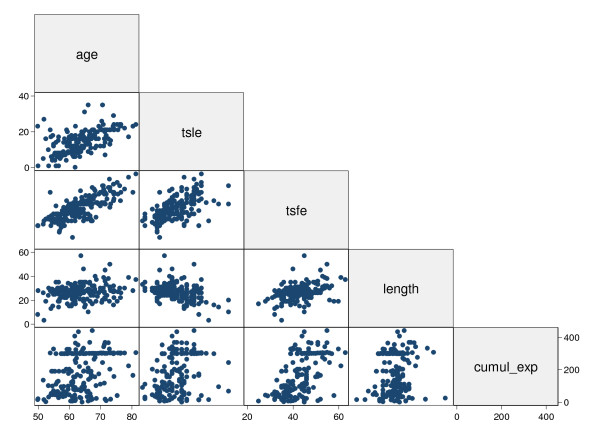
**Scatterplot matrix of the independent variables: age; time since last exposure (tsle); time since first exposure (tsfe); length of exposure; and cumulative exposure**. Simple correlation coefficient (r) in every plot.

Since they were not statistically different, data from both groups were pooled together. Figure 3 shows that age was strongly correlated with some exposure variables: TSFE (r = 0.77) and TSLE (r = 0.60). There was therefore evidence of collinearity among predictors.

Table 3 shows the regression coefficient (b), standard error of the coefficient (SE(b)), and p-value of the z test statistics provided by the linear regression analysis. At univariable analysis, OPN was found to be associated with age (positive correlation), TSLE and TSFE (positive correlation), and cumulative exposure (positive correlation). Since smoking and peak exposure were not continuous variables, the regression coefficients could be interpreted as linear trend across ordered categories. The results of multivariable analysis (Model 1 and 2) are shown in the same table 3. Only TSLE and length of exposure (positive correlation) entered the multivariable model. It can be seen that, for TSLE, SE(b) from simple regression (0.22) was fairly similar with that of Model 1 (0.23) but different from that of Model 2 (0.35). Likewise, for age, SE(b) was 0.21 at univariable analysis and 0.33 in the Model 2. The inflation of SE(b) in the Model 2 demonstrated the presence of multicollinearity. The coefficient "b" for age was 0.94 in the simple regression and 0.19 in the Model 2, revealing the presence of a strong confounding.

**Table 3:** Linear regression coefficient (b), standard error of the coefficient (SE(b)) and p-value of the z test statistics at univariable and in two models of multivariable regression

Predictor variables ^§^	Univariable analysis			Multivariable analysis					
				
				Model 1 ^@^			Model 2 ^#^		
	
	b	SE(b)	p-value	b	SE(b)	p-value	b	SE(b)	p-value
Age (years)	0.94	0.21	0.000				0.19	0.33	0.570
Time since last exposure (years)	0.83	0.22	0.000	1.13	0.23	0.000	0.98	0.35	0.005
Time since first exposure (years)	0.99	0.20	0.000						
Length of exposure (years)	0.37	0.21	0.090	0.77	0.22	0.000	0.68	0.27	0.014
Cumulative exposure (ff/ml) × years	0.03	0.01	0.009						
Peak exposure (ff/ml)	0.03	0.02	0.150						
Smoking classes	- 0.77	2.36	0.746						

^§ ^The dependent variable is Osteopontin in all models of linear regression analysis

^@ ^Estimated with forward stepwise selection of predictors

^# ^Estimated forcing age in the model 1.

Table 4 is similar to table 3, with the difference that the dependent variable was the reciprocal of OPN. It can be seen that at univariable analysis, the sign of "b" is always negative, revealing a negative correlation between 1/OPN and exposure variables (thus confirming the positive association between OPN and asbestos exposure variable). Only TSFE entered the Model 1 of multivariable analysis; it is worth noting that TSFE is the sum of TSLE and length of exposure. The SE(b) obtained at simple regression and Model 2 of multivariable analysis was, respectively, 5.50E-05 and 8.65E-5 for TSFE; 6.00E-05 and 9.28E-5 for age. There was evidence of multicollinearity, but not of strong confounding since the regression coefficients did not change markedly.

**Table 4:** Linear regression coefficient (b), standard error of the coefficient (SE(b)) and p-value of the z test statistics at univariable and in two models of multivariable regression: (1) estimated with forward stepwise selection of predictors; and (2) estimated forcing age in the former model

Predictor variables ^§^	Univariable analysis			Multivariable analysis					
				
				Model 1 ^@^			Model 2 ^#^		
	
	b	SE(b)	p-value	b	SE(b)	p-value	b	SE(b)	p-value
Age (years)	-1.95E-4	6.00E-5	0.001				1.41E-5	9.28E-5	0.879
Time since last exposure (years)	-2.28E-4	5.90E-5	0.000						
Time since first exposure (years)	-2.42E-4	5.50E-5	0.000	-2.42E-4	5.47E-5	0.000	-2.5E-4	8.65E-5	0.004
Length of exposure (years)	-1.06E-5	5.90E-5	0.858						
Cumulative exposure (ff/ml) × years	-7.87E-6	3.25E-6	0.016						
Peak exposure (ff/ml)	-1.09E-5	6.41E-6	0.089						
Smoking classes	-2.79E-4	6.50E-4	0.668						

^§ ^The dependent variable is Osteopontin in all models of linear regression analysis

^@ ^Estimated with forward stepwise selection of predictors

^# ^Estimated forcing age in the model 1.

The dependent variable is 1/Osteopontin in all models of linear regression analysis.

## Discussion

We aimed to assess an association between plasma levels of OPN and, on the other hand, asbestos exposure or the presence of asbestos related benign diseases. This study suggests that OPN is not a reliable marker for the presence of pleural plaques (table 1,2; and figure 1,2): if OPN were a profibrotic mediator of some lung compartments [9], there would be different levels in subjects with or without PP. Likewise, a recent study [10] reported that the average level of OPN in healthy subjects exposed to asbestos (n = 112) was not significantly different from that in subjects with benign asbestos disease (n = 33).

The present study also suggests that OPN is not a reliable biomarker of the lung burden of asbestos since an increasing relation was found between OPN and TSLE (table 3). However, it is known that chrysotile dissolves fairly rapidly in lung tissue - the half-life is approximately six months and some papers suggest that this time period may be even shorter [11-13]. Pulmonary amphiboles are also removed but at a slower rate. It has been calculated that in humans the elimination of crocidolite fibres is 10 to 15% per year, which means that every 7-10 years half of the contents of fibres accumulated in the lung will be destroyed and/or expelled [14]. Since the median TSLE was about 15 years (table 1), in about 50% of examined workers the lung burden should be approximately zero for chrysotile and reduced by more than half for amphiboles.

Age was found to be a strong confounder and a source of multicollinearity (Model 2 in table 3 and 4) since it was significantly and positively correlated with OPN (tables 2, figure 1) and the time indicators of asbestos exposure (figure 3). Deleting age from the model (as in Model 1 of tables 3 and 4) greatly reduced the standard error; but the increase in the precision of estimate may be offset by an increase in bias due to inadequate control of confounding [15].

According to Pass [5], serum OPN levels were significantly (p < 0.02) higher in workers with more than 10 years as compared to those with 0-9 years of exposure. Pass [5] also reported that in patients with "plaques and fibrosis" serum OPN was significantly (p < 0.004) higher than in subjects with normal or "other" radiographic findings. Due to the similarity of the points shown in a graph (figure 3 of the latter paper), workers with plaques and fibrosis also had more than 10 years of asbestos exposure. However, the present study envisages the possibility that these findings, at least in part, might be the result of confounding by age or have arisen by chance.

One could ask if there is any reason for assessing OPN levels only in plasma and not in serum. The reason is related to evidence that differences between serum and plasma samples exist for measurement of OPN. OPN present in whole blood is cleaved in the course of blood coagulation and cleavage by thrombin can produce OPN fragments that have biological activity different from the whole protein [16]. Therefore, it is not recommended to use serum or heparin plasma in the OPN assay as this protein is likely to be cleaved in these matrices. Moreover, plasma was the preferred clinical specimen for measurement of OPN in studies for diagnosis of epithelial malignant pleural mesothelioma because OPN serum levels were influenced by pre-analytical factors like as thawing [17]. Lastly, other than the choice of material, e.g. plasma vs. serum, the duration of storage may also affect the results of the OPN measurement by ELISA [18].

In our population the median OPN plasma level was 49.9 ng/ml, whereas a previous study has shown OPN plasma levels of 28.3 ng/ml in subjects with non-malignant pulmonary diseases [19]. The discrepancy could be attributed to the different OPN ELISA commercial kits used in the two studies (Assay Designs and R&D Systems, respectively). A systemic bias was also found between human osteopontin ELISA commercial kits supplied by different manufactures (Immuno-Biological Laboratories Co., Ltd, Gunma, Japan *vs *R&D systems, Minneapolis, MN, USA), with lower concentrations measured by R&D systems assay [20]. Moreover, a review of the literature suggests that different commercially available OPN ELISA systems produce different absolute plasma OPN levels and that even with identical ELISA systems plasma OPN levels measured can only be compared with caution [18].

Some limitations of the study must also be discussed. The first limitation of the study is the lack of cases of asbestosis. Another limitation comes from the fact that, since PP cases were relatively few, 8 subjects with PP not present in the original list and lately addressed to the OHS were added to the sample. This fact may have limited the representativeness of the sample. However, the issue with this study was analytical (is OPN associated to asbestos exposure or to the most common benign asbestos disease - PP?) rather than descriptive (which is the distribution of OPN levels in the source population); only in the latter event the sample should be strictly representative of the large base population. A third limitation is the lack of an objective measure of asbestos concentrations over time. The past exposure was estimated retrospectively, generally using the JSM approach. However, in 772 asbestos workers examined with computed tomography, past exposure (estimated with the above method) was strongly related with the risk of benign asbestos disease (p trend <0.001), demonstrating that the assessment of the historical asbestos exposure was valid [7].

## Conclusions

Plasma Osteopontin cannot be used as surrogate of asbestos exposure and does not differentiate asbestos workers with or without pleural plaques. Therefore, in the absence of actual exposure measurements, the best method for determining historical exposure to asbestos is the JSM or JEM approach.

## Competing interests

The authors declare that they have no competing interests.

## Authors' contributions

GM (first author) designed the study, performed the statistical analysis and contributed to the drafting of the paper. GM (second author) designed the study and coordinated the research team. LC participated in the statistical analysis and contributed to the drafting of the paper. SM, ASCF and MG carried out the laboratory tests, provided technical support, and contributed to the drafting of the paper. MNB and FV provided technical and logistic support. UF provided epidemiological support. JHL contributed to the drafting of the paper. All authors read and approved the final version of the manuscript.

## Pre-publication history

The pre-publication history for this paper can be accessed here:

http://www.biomedcentral.com/1471-2458/11/220/prepub
